# The Sexual Dimorphism of Lipid Kinetics in Humans

**DOI:** 10.3389/fendo.2015.00103

**Published:** 2015-07-02

**Authors:** Sylvia Santosa, Michael D. Jensen

**Affiliations:** ^1^Department of Exercise Science, Concordia University, Montreal, QC, Canada; ^2^Nutrition, Obesity, and Metabolism Laboratory, PERFORM Centre, Montreal, QC, Canada; ^3^Endocrine Research Unit, Mayo Clinic, Rochester, MN, USA

**Keywords:** sex steroids, estrogen, testosterone, sex hormone, metabolism, fatty acid, lipids

## Abstract

In addition to the obvious differences in body shape, there are substantial differences in lipid metabolism between men and women. These differences include how dietary fatty acids are handled, the secretion and clearance of very low-density lipoprotein-triglycerides, the release rates of free fatty acids (FFA) from adipose tissue relative to energy needs, and the removal of FFA from the circulation, including the storage of FFA into adipose tissue via the direct uptake process. We will review what is known about these processes and how they may contribute to the sexual dimorphism of body fat distribution.

## Introduction

Where we store fat in our bodies is a significant predictor of metabolic disease risk. Metabolic diseases predominantly affect people with an upper body/android fat distribution compared to those with a lower body/gynoid distribution (1). Jean Vague was the first to report the relationship between “android” obesity and diabetes (2). Since that report, numerous studies have confirmed the relationship between central adiposity and risk of diabetes (3, 4), cardiovascular disease risk and events (5, 6), hypertension (7), sleep apnea (8), cancer (9), and overall mortality rates (10); men and women in the highest quintile of measured waist circumference (WC) are reported to have almost twice the mortality rates than those in the lower quintiles. Though we do not fully understand why people are shaped differently, one of the most obvious factors determining body shape is sex hormone status.

Healthy, modestly active/sedentary women with BMIs of 20–25 kg/m^2^ often have 25–35% body fat, whereas men of the same BMI have 10–20% body fat; highly athletic women and men can have less body fat (11). Typical non-obese adult males have little leg fat relative to upper body fat and most non-obese, premenopausal adult females have ample lower body fat stores and limited visceral adipose tissue (VAT). In contrast, men have greater amounts of VAT, even controlling for body fat mass, and VAT constitutes a greater proportion of total fat mass in men than women, especially at higher BMIs (12). The mechanisms underlying the sexual dimorphism in body fat and its distribution between men and women are not well understood. Studies that investigate the contribution of lipid metabolism to body shape indicate a staunch effect of sex steroids. The objective of this review is to examine how lipid metabolism contributes to differences in body fat distribution and the role of sex hormones in modulating these differences.

## Fatty Acid Metabolism

The principle function of adipose tissue is to store and release FA in response to changes in energy balance. Following consumption of a fat containing meal, a significant proportion of circulating chylomicron-triacylglycerol (TAG) fatty acids (FA) is stored via lipoprotein lipase (LPL) into adipose tissue. In this pathway, endothelial LPL hydrolyzes FA from the glycerol backbone of chylomicron-TAG, allowing them to enter the adjacent adipose tissue. Some of the FAs from chylomicron-TAG are not taken up locally, but instead “spillover” into systemic circulation. A less appreciated pathway of FA storage occurs via the direct re-uptake of free fatty acids (FFA) from the systemic circulation independent of the LPL mechanism. Although we typically envision that the FFA released as a result of adipose tissue lipolysis are destined to supply fuel to lean tissues for fat oxidation, a portion is taken up and stored in distant adipocytes. We have referred to this process as direct FFA storage.

In addition to LPL, there are a number of other proteins and enzymes that are involved in adipose tissue FA metabolism. When it comes to FA entry into cells, FA can be taken up by passive (flip–flop) diffusion or via membrane protein facilitated diffusion (13). One of the more closely examined proteins involved in facilitated diffusion is the FA transport protein CD36. CD36 is a cell surface glycoprotein that is particularly important when extracellular FA concentrations are reduced (13, 14). CD36 appears to play a main role in the localization of long chain FA to the adipocyte membrane through FA binding (13). Recent studies indicate that CD36 facilitates muscle and adipose tissue FFA uptake under conditions of reduced FFA availability, whereas hepatic FFA uptake appears to be CD36-independent and myocardial uptake is partially CD36-dependent across a wider range of FFA concentrations (14). Other proteins that have been investigated in their role of FA storage are acyl-CoA synthetase (ACS) and diacylglycerol acyl transferase (DGAT). ACS catalyzes the intracellular activation of the FA to their CoA form (15). DGAT, the final step of storage of FA as TAG, is suggested to be a rate limiting enzyme in FA storage (16). There are three enzymes involved in triglyceride hydrolysis: adipose triglyceride lipase (ATGL), hormone-sensitive lipase (HSL), and monoglyceride lipase (MGL) (17). Of these three enzymes, HSL has been the most studied and has been linked to aberrations in lipolysis associated with obesity (18). HSL hydrolyzes an assortment of substrates including TAG, diacylglycerol, and monoacylglycerol (17). We have postulated that the activity of proteins involved in FA storage and lipolysis play key roles in modulating regional fat distribution.

The relative and absolute size of the different adipose tissue depots is ultimately determined by the balance between lipolysis on one hand and storage of FA from chylomicrons, very low-density lipoprotein (VLDL), and direct FFA uptake on the other. Although other factors, such as the gain or loss of lean tissue and changes in endocrine signaling, impact body composition, this review will focus on studies that examine the contributions of FA metabolism in determining regional body composition. Several studies have been conducted to expand our understanding of how the variation in body fat distribution in men and women relate to systemic and regional fat metabolism.

## Lipid Metabolism in Men and Women

**Figure 1:**
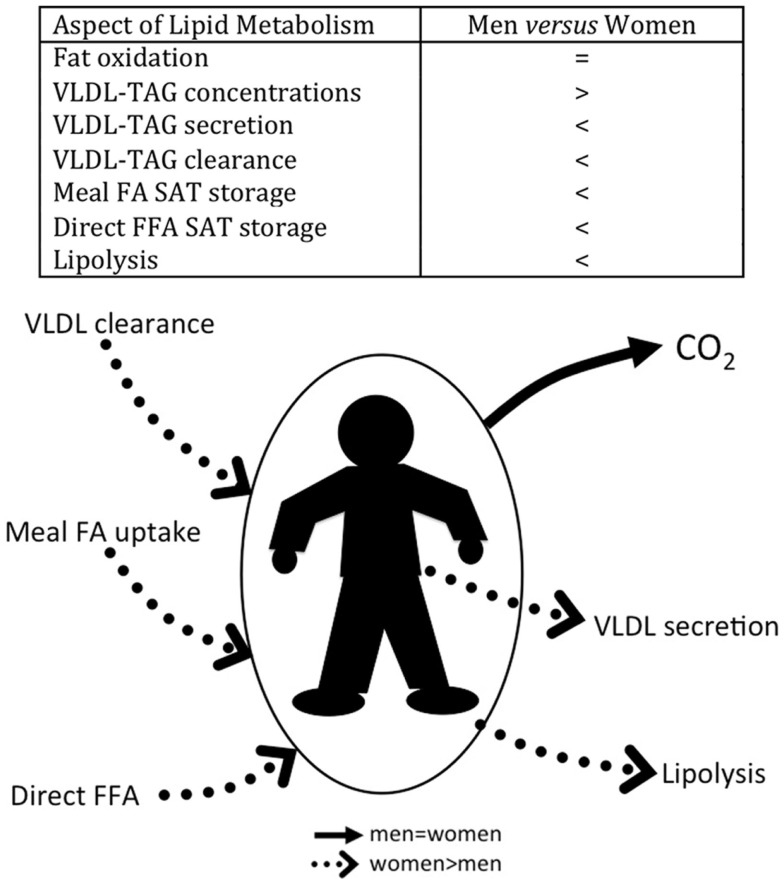
**Summary of relative FFA metabolism in young, lean, fasted men, and age- and BMI-matched fasted premenopausal women**.

Figure 1 summarizes the findings in lean men vs. age- and BMI-matched premenopausal women for each aspect of FA metabolism discussed below.

### Fatty acid oxidation

In the postprandial and postabsorptive state, FA oxidation does not appear to be different between men and women whereas during exercise fat oxidation is higher in women. This is because there are several factors other than sex that are potential independent contributors toward the variations in fat oxidation between individuals including energy balance, age, heredity, and body composition (19). Because fat oxidation has been shown to relate to fat free mass (FFM), and because men and women of the same weight often differ with regards to body composition, comparing fat oxidation in milligrams per minute or milligram per kilogram per minute may be misleading. In determining whether there are differences in fat oxidation between sexes, FFM can be accounted for by statistical adjustment or fat oxidation can be expressed in the context of total energy expenditure (as RQ or a percent). Unless men and women are matched for FFM, we prefer not to express substrate oxidation divided by FFM per unit time. This is because the relationship between energy expenditure and FFM is not ratio-standard; that is, the intercept is not zero and the slope is not 1. On average at a given energy expenditure (substrate oxidation), individuals with lower FFM will *de facto* have greater energy expenditure per kilogram FFM than those with greater amounts of FFM.

When the results of tracer studies were integrated, it was suggested that over a day that includes 2 h of exercise, 14 h in the postabsorptive state, and 8 h in the postprandial state, fat oxidation was similar in men and women (20). This conclusion was supported by a study that measured 24 h RQ in a whole room calorimeter that included spontaneous physical activity, which found no differences between men and women (19). The lack of a difference in fat oxidation between men and women remains after adjusting for FFM during rest (21) or during a 24 h period with the incorporation of light–moderate physical activity (22). However, it should be noted that it is difficult to draw conclusions based on these and the following studies discussed herein for lack of appropriate statistical adjustment for FFM. In contrast, a whole room calorimeter study by Melanson et al. (23) found that women had 24–56% greater 24 h fat oxidation (milligram fat oxidation per kilogram FFM) than men, regardless of physical activity levels. Women in the study also had an enzymatic profile that favored β-oxidation in muscle (23). A review by Henderson (24) concluded that men oxidize more fat than women in the post-exercise state provided that they remain fasting. However, if food is ingested, this difference is no longer present. Additionally, with aging, changes in sex steroid concentrations may impact fat oxidation, as fat oxidation (rather than glucose oxidation) was observed to decrease in proportion to energy expenditure (21). Hober et al. (25) showed that with increasing age in males, resting fat oxidation (gram per kilogram lean mass) decreases whereas in females, no relationship was observed between resting fat oxidation and age. Thus, fluctuations in sex hormones as a result of age may be partially responsible for differences in fat oxidation between men and women.

### Very low-density lipoprotein TAG kinetics

In the fasting state, most circulating plasma TAG are in VLDL, which are synthesized and secreted by the liver (26). Similar to chylomicron-TAG, VLDL-TAG are hydrolyzed by LPL residing on the capillary endothelium, making FA available to the underlying tissue for energy or storage. It is well established that men have higher circulating VLDL-TAG than age-matched women (27), but the etiology of this sex difference is not completely understood. Lean women have both greater VLDL-TAG secretion and clearance compared with lean men (28), which causes us to favor greater relative removal of VLDL-TAG as the primary explanation. In obesity, increased rates of VLDL-TAG secretion and decreased rates of VLDL-TAG clearance in men vs. women have been reported (28, 29).

### Meal fatty acid storage

Patterns of meal FA storage only somewhat mirror the patterns of regional fat distribution. We initially found no differences in patterns of meal FA storage in men and women; more meal FAs were stored (per gram lipid) in upper body SAT than in leg SAT in both groups (30–32). However, following consumption of a high-fat meal, women stored a greater proportion of meal fat in lower body SAT whereas men did not (33). Moreover, when meal FA storage was compared in men and women with different obesity phenotypes, variations in regional meal fat storage were found (34). In women with lower body obesity, meal FA storage was greater in gluteal than abdominal fat (34), whereas storage was similar in these two depots in upper body obese men and women. Furthermore, upper body obese men stored a lesser percent of meal fat in subcutaneous fat than either group of women (34). This suggests that meal FA storage may play a role in determining the sex differences in regional adiposity.

### Direct FFA storage

Even more so than meal FA storage, patterns of circulating FFA storage (termed, “direct FFA storage”) correspond to the sexual dimorphism in fat distribution. When the direct FFA storage pathway was first reported, FFA storage into SAT was consistent with body shape in men and women. More specifically, direct FFA storage in SAT was less in men than women, and men stored more FFA in abdominal vs. femoral SAT depots whereas women did not (35). Follow-up studies confirmed these results and noted that FFA storage rates were greater in femoral than abdominal SAT in women (36). In women with varying regional fat distribution, direct storage of FFA also followed regional fat distribution (37). These studies support a role of direct FFA storage as a means of redistributing FAs in a manner that appears to influence body shape.

### Lipolysis

If rates of lipolysis were a major contributor toward sex differences in AT deposition, we would expect sex differences in the relative rates of total and regional lipolysis in men compared to women. For example, since women have greater lower body fat mass, lower rates of leg adipose tissue lipolysis would be observed in women compared with men. However, we found that the opposite is true. We found that leg adipose tissue lipolysis rates were 44 μmol/min in lean and 107 μmol/min in obese men, and 61 μmol/min in lean and 107 μmol/min in obese women. Thus, lipolysis in leg SAT of men and women was approximately equal, and even a little higher in lean women vs. men (38). Moreover, studies collectively show that regardless of sex, upper body AT is more lipolytically active than lower body (38–42). Therefore, lipolysis does not appear to explain the sexual dimorphism seen in body fat distribution.

## The Effect of Estrogen and Testosterone on Lipid Metabolism

The sexual dimorphism in adiposity along with differences in fat metabolism found in men and women allude to a compelling role of sex steroids in determining regional fat distribution. Several studies have examined the effects of sex steroid suppression and/or supplementation on fat metabolism. Table 1 summarizes what is known about the changes in lipid metabolism that occurs with sex steroid suppression in men and women.

**Table 1:** **Changes in lipid metabolism as a result of estrogen deficiency in women and testosterone deficiency in men relative to eugonadal women and men, respectively**.

	E	T
Fat oxidation	↓	↓
VLDL-TAG	↑	=
VLDL-TAG secretion	↓ ↓	=
VLDL-TAG clearance	?	?
Abdominal meal FA storage	=	=
Leg meal FA storage	↑ ↑	↑
Direct FFA SAT storage	↑	=
Lipolysis	↑	=

*All symbols compare deficient to eugonadal individuals. ↑ or ↓, increases or decreases; ↑ ↑ or ↓ ↓, large increases or large decreases; =, no differences between deficient and eugonadal individuals; ?, unclear findings with regards to the effects of sex steroid deficiency*.

### Fat oxidation

Periodic 24 h measurements over a 4-year period showed that fat oxidation did not change in premenopausal women, whereas in postmenopasual women, fat oxidation decreased 32% (43). However, significant changes in body composition and diet may have affected the changes in fat oxidation in these women (43). We were able to exclude differences in body composition as the factor influencing fat oxidation in pre- vs. postmenopausal women by studying two groups of women matched for age, BMI, and body fat (44). We found that postprandial fat oxidation was indeed lower in postmenopausal women (44), confirming that hypogonadism/estrogen deficiency reduces fat oxidation, at least during a portion of the day. Whether the opposite is true, i.e. whether estrogen repletion increases fat oxidation, is unclear. When transdermal estrogen is provided to postmenopausal women over 6 months, neither changes in body composition nor changes in fat oxidation were observed (45). However, another study that supplemented estrogen transdermally over a 12-month period observed increases in lipid oxidation. These women had an increase in lean mass and no changes in fat mass over the 12-month estrogen repletion period (46). The different results in these studies could stem from the changes in body composition that occurred over the treatment period. More studies are required to tease out whether estrogen repletion in women affects fat oxidation.

We believe the balance of data suggests that testosterone increases fat oxidation in men. One study showed that acute hypogonadism did not change fat oxidation over a 3-week period compared to men replete with estrogen and/or testosterone (47). In contrast, Host et al. (48) showed that 1 month of hypogonadism decreased fat oxidation. This finding was supported by another study, which observed that hypogonadism over 10 weeks decreased rates of fat oxidation; however, decreases in fat free and increases in fat mass may partially explain these changes (49). To address the potential effect of body composition on fat oxidation, we matched chronically hypogonadal males for age and body composition and found lower fat oxidation in hypogonadal vs. eugonadal males (50). Four months of testosterone treatment in adolescent males with delayed puberty resulted in an increase in fat oxidation (51). Although 2 years of transdermal testosterone supplementation in elderly men with modestly reduced testosterone did not change body composition or meal fat oxidation, these men were not truly hypogonadal (52).

### VLDL-TAG kinetics

Studies examining the effects of sex hormones on VLDL-TAG kinetics in men and women do not appear to clarify the lower VLDL-TAG concentrations in women. VLDL-TAG secretion in premenopausal women was found to be twice that of postmenopausal women despite higher VLDL-TAG concentrations in postmenopausal women (29). In another study, women treated with estradiol vs. progesterone vs. testosterone vs. control were compared (53). VLDL-TAG kinetics did not change with placebo, or progesterone or testosterone treatment but clearance increased in the estrogen-treated group in conjunction with decreased VLDL-TAG concentrations (53). There were no differences in VLDL-TAG secretion in any of the groups of the study (53). The finding that testosterone supplementation in women did not affect VLDL kinetics was consistent with that of another study by the same group that supplemented testosterone in obese women for 3 weeks (54). In men, 1 month of hypogonadism and acute testosterone repletion to physiological levels did not affect VLDL-TAG secretion and concentrations (48).

### Meal FA storage

Few studies have been conducted to examine the effects of estrogen on meal FA storage. We found that postmenopausal had greater meal FA storage than premenopausal women (44). We also found that, compared to premenopausal women, meal FA storage in the femoral depot was two times greater in postmenopausal women (44). Another study that used LPL as a surrogate for meal FA storage observed that in postmenopausal women treated with estrogen and progesterone, LPL activity was higher in femoral vs. abdominal adipocytes (55). These studies indicate that estrogen affects meal FA storage in such a way as to promote meal fat storage in lower body fat in women. Why postmenopausal women would thus tend to gain abdominal fat is unclear.

There have been a limited number of studies on the effects of acute or chronic testosterone hormonal manipulation on meal FA metabolism. We found that chronically hypogonadal men stored more meal FA in the femoral region than eugonadal men (50). Whereas meal FA storage (milligram per gram lipid) in hypogonadal men was similar in the abdominal and femoral region, eugonadal men tended to store more meal FA in the abdominal than thigh region (50). These patterns of fat storage would be consistent with what we would expect given the regional differences in fat distribution between hypogonadal and eugonadal men. Thus, testosterone likely affects regional adiposity through its effects on regional meal FA storage. We did find that 2 years of testosterone supplementation of older men with somewhat reduced testosterone concentrations increased meal FA storage in the abdominal vs. femoral region (52). In another study, acute (5 days) of testosterone treatment decreased meal FA storage in VAT (30). In contrast, Marin et al. (31) found at baseline that more meal FA was stored in the abdominal vs. femoral depot and that after 9 months of testosterone treatment, these differences in meal FA storage no longer existed (31). This shift was likely because of a significant decrease in meal FA storage in abdominal SAT with T treatment. Potential reasons for this contradictory finding could stem from differences in ages of men between studies, differences in the testosterone treatment period, or differences in the timing of biopsies after ingestion of the experimental meal (56).

### Direct FFA storage

We matched pre- and postmenopausal women for age and body composition and found that direct FFA storage was greater in postmenopausal than premenopausal women (44). However, we found no significant regional differences in the storage of FFA between these two groups (44). A closer examination of the key factors involved in FFA storage showed that activity of ACS and DGAT were significantly upregulated in postmenopausal compared to premenopausal women indicating greater potential for FFA storage in postmenopausal women. In a second study, we found no differences in the rates of FFA storage between age and body composition match men who were chronically hypogonadal and eugonadal (50). However, activity of ACS was significantly greater in femoral adipose tissue of eugondal than hypogonadal men. The effects of sex steroids on direct FFA storage remains to be investigated before a conclusion can be drawn.

### Lipolysis

Although regional differences in the relative rates of lipolysis are unlikely to be central in determining regional fat differences in men and women, there are major differences in rates of lipolysis relative to energy expenditure (38, 39). The rates of FFA release are ~40% greater in women than men relative to energy needs (38), despite similar relative rates of fat oxidation. Studies that manipulate sex steroids in humans support this conclusion. Contrary to what would be expected, decreases in sex steroids increase lipolysis whereas no differences in lipolysis occur with hormone treatment. Estrogen deficiency in women resulted in a 10–20% increase in lipolysis whereas estrogen repletion did not affect adrenergic stimulated lipolysis (57). This finding is consistent with that of another study, which showed that estrogen and progesterone treatment in postmenopausal women did not affect lipolysis (55).

Testosterone treatment has been shown to decrease total and abdominal fat mass and thus, would be expected to increase lipolysis. Testosterone treatment has been shown to stimulate lipolysis in the abdominal region (55) and in VAT (58). However, these cited studies involved shorter treatment periods. Another short-term study found that 7–10 days of hypogonadism and acute testosterone rescue did not have any effects on indicators of adipose tissue lipolysis (48). These findings are consistent with a longer 2-year period of testosterone treatment in hypogonadal men that did not detect any changes in lipolysis from upper or lower body SAT (52). Moreover, whole-body lipolysis was also found to be unchanged in adolescent males after 4 months of testosterone treatment (51).

## Conclusion

The objective of this review was to describe the differences in lipid metabolism between males and females and to link, when possible, these differences to how they may cause or create the differences in body shape. Subsequently, we also sought to explore how sex steroids regulate this relationship. Fat oxidation, lipolysis, VLDL-TAG, meal FA metabolism, and direct FFA storage may collectively contribute toward total and regional adiposity. Several studies have been conducted to examine these aspects of fat distribution in the context of differences in sex and sex steroids. Regional meal FA and direct FFA storage appear to contribute toward the sexual dimorphism in fat distribution. While the extent to which fat oxidation and VLDL-TAG kinetics contribute to body composition are unclear, it is evident that lipolysis has little effect in contributing toward differences in body shape between men and women.

## Conflict of Interest Statement

The authors declare that the research was conducted in the absence of any commercial or financial relationships that could be construed as a potential conflict of interest.
